# Novel Synthetic Lipopeptides as Potential Mucosal Adjuvants Enhanced SARS-CoV-2 rRBD-Induced Immune Response

**DOI:** 10.3389/fimmu.2022.833418

**Published:** 2022-03-09

**Authors:** Ling Mao, Chang Liu, Jing-Yi Liu, Zi-Li Jin, Zhe Jin, Ruo-Yi Xue, Rang Feng, Guo-Cheng Li, Yan Deng, Hao Cheng, Quan-Ming Zou, Hai-Bo Li

**Affiliations:** National Engineering Research Center of Immunological Products, Department of Microbiology and Biochemical Pharmacy, College of Pharmacy, Third Military Medical University, Chongqing, China

**Keywords:** lipopeptide, vaccine adjuvant, structure-activity relationship, SARS-CoV-2 rRBD, support vector machine

## Abstract

As TLR2 agonists, several lipopeptides had been proved to be candidate vaccine adjuvants. In our previous study, lipopeptides mimicking N-terminal structures of the bacterial lipoproteins were also able to promote antigen-specific immune response. However, the structure-activity relationship of lipopeptides as TLR2 agonists is still unclear. Here, 23 synthetic lipopeptides with the same lipid moiety but different peptide sequences were synthesized, and their TLR2 activities *in vitro* and mucosal adjuvant effects to OVA were evaluated. LP1-14, LP1-30, LP1-34 and LP2-2 exhibited significantly lower cytotoxicity and stronger TLR2 activity compared with Pam_2_CSK_4_, the latter being one of the most potent TLR2 agonists. LP1-34 and LP2-2 assisted OVA to induce more profound specific IgG in sera or sIgA in BALF than Pam_2_CSK_4_. Furthermore, the possibility of LP1-34, LP2-2 and Pam_2_CSK_4_ as the mucosal adjuvant for the SARS-CoV-2 recombinant RBD (rRBD) was investigated. Intranasally immunized with rRBD plus either the novel lipopeptide or Pam_2_CSK_4_ significantly increased the levels of specific serum and respiratory mucosal IgG and IgA, while rRBD alone failed to induce specific immune response due to its low immunogenicity. The novel lipopeptides, especially LP2-2, significantly increased levels of rRBD-induced SARS-CoV-2 neutralizing antibody in sera, BALF and nasal wash. Finally, Support vector machine (SVM) results suggested that charged residues in lipopeptides might be beneficial to the agonist activity, while lipophilic residues might adversely affect the agonistic activity. Figuring out the relationship between peptide sequence in the lipopeptide and its TLR2 activity may lay the foundation for the rational design of novel lipopeptide adjuvant for COVID-19 vaccine.

## Introduction

Vaccination is one of the most influential events in medical history, which could decrease the mortality of many diseases and improve the quality of life (1). Actually, vaccination is a process that mimics natural infection to generate a potent protective immune response by activating immune system (2). As a result of research and development efforts, diverse types of vaccines have been authorized, such as recombinant subunit protein vaccine, polysaccharide conjugate vaccine and peptide vaccine (3–5). However, these vaccines are limited because of poor immunogenicity (6). Therefore, it is urgent to find new methods to strengthen the efficacy of vaccines, such as adding adjuvants.

Adjuvants are the materials that have been added into vaccines to enhance immune response. They may reduce the frequency of booster immunizations, shape the adaptive immune response, decrease the dose of antigen and improve the protective efficacy (7). Toll-like receptors (TLRs) play essential roles in innate immune responses against microbial pathogens, as well as the subsequent induction of adaptive immune responses (8). Based on the important roles of TLRs in immune response, TLRs agonists have been reported as promising adjuvants in vaccines (9, 10), such as TLR4 agonist MPL (11) and TLR9 agonist CpG ODN (12), which have been included in the licensed vaccines. TLR2 forms a heterodimer with TLR1 or TLR6, the corresponding ligands could bind with them thereby inducing inflammatory cytokines release (13). It is well known that lipopeptides containing Pam_2_Cys and Pam_3_Cys could activate TLR2 signaling (14) and they are widely used in adjuvant studies (15, 16). Pam_2_CSK_4,_ one of the most potent TLR2 agonists, was a Th2 polarizing adjuvant in murine models (17).

In recent years, a variety of lipopeptides have been designed and synthesized to exert adjuvant effects through activating TLR2 (18–20). In our previous study, lipopeptides containing N-terminal sequences of the bacterial lipoproteins had been shown to promote antigen-specific immune response (21). Though those lipopeptides possess the same lipid moiety, FSL-1 and FSL-2 differs in only one amino acid at the C-terminus of the peptide portion, and the TLR2 activity of FSL-2 is only 80% that of FSL-1 (22). The TLR2 activity of FSL-1 can reach 4 times that of MALP-2, while Pam_2_CSK_4_ is the most potent TLR2 agonist among the four lipopeptides (23). It has been recognized that the peptide sequence in the lipopeptide has a significant effect on its TLR2 activity, but how the peptide sequence affects its activity is not fully understood.

Vaccine-mediated mucosal response plays crucial roles in prevent infection at the portal of entry (24). As TLR2 agonists, some lipopeptides have been proved to be the potent mucosal adjuvants. Intranasal vaccination using a lipopeptide as the adjuvant was able to induce elevated production of IgG in serum, sIgA in bronchoalveolar lavage fluid (BALF) (25). However, whether the lipopeptide could enhance the efficacy of COVID-19 vaccine remains unknown. In this study, 23 novel synthetic lipopeptides were synthesized and their TLR2 agonistic activities were evaluated. LP1-30, LP1-34, LP2-2 and LP2-3 possess more potent TLR2 agonistic activity compared with Pam_2_CSK_4_. Mice were immunized with the mixture of the four novel synthetic lipopeptides and OVA through intranasal routes, and the levels of specific sIgA in almost all mucosal sites were determined. In addition, the mucosal adjuvant effects of LP1-34 and LP2-2 to recombinant RBD (rRBD), a COVID-19 subunit vaccine candidate, were evaluated. Finally, Support vector machine (SVM) was used to investigate the influence of peptide sequence on the TLR2 activity of the lipopeptides.

## Materials and Methods

### Reagents

DMEM (C11995500BT), Fetal bovine serum qualified Australia (10099141C) and Trypsin-EDTA (0.25%), phenol red (25200072) were purchased from Gibco; CD11c-FITC (117306), CD86-PerCP (105026), CD80-APC (104714) and CD40-PE (124610) were purchased from Biolegend; Goat Anti-Mouse IgG Antibody (HRP) (ab98762), Goat Anti-Mouse IgA alpha chain (HRP) (ab97235), Goat Anti-Mouse IgG1 heavychain (HRP) (ab97240), Goat Anti-Mouse IgG2a heavy chain (HRP) (ab97245), TMB ELISA Substrate (ab171523) and 450nm stop solution for TMB substrate (ab171529) were purchased from Abcam; Penicillin-Streptomycin Solution100X (C0222), and BSA, FranctionV (ST023) were purchased from Beyotime; Mouse IFN-gamma ELISpot PLUS kit (HRP) (3321-4HPW-2), Mouse TNFa ELISA kit (1217202), Mouse IL-1β ELISA kit (1210122), and Mouse IL-12p70 ELISA kit (1211202) were purchased from Dakewe; GM-CSF (315-02-10ug) and IL-4 (214-14-20ug) were purchased from PEPROTECH; Quant-iTTM PicoGreendsDNA Reagent (P7581) was purchased from Thermo Scientific; 2019-nCoV Protein RBD (DRA36) was purchased from novoprotein; Albumin from chicken egg white (A5503) was purchased from sigma.

### Ethics Statement

Animal maintenance and laboratory procedures were performed in accordance with the Regulations for the Administration of Affairs Concerning Experimental Animals approved by the State Council of People’s Republic of China. All animal experiments were approved by the Animal Ethical and Experimental Committee of the Third Military Medical University. Well-trained and skilled animal care personnel participated in the current study to minimize the suffering of animals.

### Mice and Cell Lines

6-8 weeks old specific-pathogen-free female BALB/c mice were purchased from the Experimental Animal Center of Army Medical University. HEK-Blue™ mTLR2 cells were purchased from *In vivo*Gen (San Diego, USA) and cultured in DMEM supplemented with 10% FBS, L-glutamine (2mM), Normocin (100μg/mL), penicillin (50U/mL) and streptomycin (50g/mL) at 37°C in 5% CO_2_.

### Synthesis of Lipopeptides

Fmoc-Pam_2_Cys was synthesized using protocols described in the **Supplementary Information**. ^1^H NMR for Fmoc-Pam_2_Cys was provided (**Figure S1**). All the lipopeptides in this study were synthesized by the method previously described (21). HPLC analysis and mass spectrometry are included in the Supplementary Information (**Figure S2**).

### TLR2 Signaling Assay

HEK-Blue™ detection was prepared according to manufacturer’s instruction. Briefly, the lipopeptides were added into a flat-bottom 96-well plate (20μL per well) and endotoxin-free water was used as a negative control. Then HEK-Blue™ mTLR2 cells were suspended in HEK-Blue™ detection and added into the plate (180μL per well, 5×10^4^ cells). After incubation at 37°C for 14h, the SEAP activity was measured by a spectrophotometer at 620nm.

### RNA Extraction and Real-Time RT-PCR

Total RNA was extracted by using RNAiso Plus (TakaRa, 9108) according to manufacturer’s instruction. Reverse transcriptional (RT)-PCR was performed by using PrimeScript RT reagent Kit (TakaRa, RR037A) according to manufacturer’s instruction. Then cDNA was quantified by SYBR green-based real-time PCR (TOYOBO, QPK-201). The following primers was used:

TLR1 forward: 5’- ACGGGTAAGGTTGTCTTGACG-3’, reverse: 5’-CTTCCGCTCTCTTCATGCCT-3’;
TLR2 forward: 5’-AAGGAGGTGCGGACTGTTTC-3’, reverse: 5’-CCGGTGATGCAATTCGGATG-3’;
TLR3 forward: 5’-GCGCATATCACAGGCTGAA-3’, reverse: 5’-ATCTTCTTTTGGTGCGCGAT-3’;
TLR4 forward: 5’-AGATCTGAGCTTCAACCCCTT-3’, reverse: 5’-GTCTCCACAGCCACCAGATT-3’;
TLR5 forward: 5’-GTATGCACTGTCACTCTGACTCTGT-3’, reverse: 5’-AGCCCCGGAACTTTGTGACT-3’;
TLR6 forward: 5’-TCGGAGAAGGAAGTCTTGAGC-3’, reverse: 5’-TATTAAGGCCAGGGCGCAAA-3’;
TLR7 forward: 5’-TGGCTCCCTTCTCAGGATGA-3’, reverse: 5’-ATGTCTCTTGCTGCCCCAAA-3’;
TLR8 forward: 5’-TGTGAGCTGAAGCCTCATGG-3’, reverse: 5’-GGACAGGTGGACGAAGTCAG-3’;
TLR9 forward: 5’-TCTGCTCTCTGCACAGCTAC-3’, reverse: 5’-CATGACTGAGGGGGCATGTT-3’;
β-actin forward: 5’-TGGCTCCTAGCACCATGAAG-3’, reverse: 5’-AACGCAGCTCAGTAACAGTC-3’.

β-actin was used as internal reference, the expressions of genes were calculated by using the comparative threshold cycle (Ct) method.

### Hemolysis Assay

Hemolysis assay was performed using protocol described previously (21). Briefly, different concentrations of each lipopeptide were mixed with 2% red blood cells, and the resulting suspension were added into a flat-bottom 96-well plate (200μL per well), using PBS as a negative control and sterile water as a positive control. After incubation at 37°C for 3h, the hemolytic activity was determined by measuring the absorbance of the supernatant at 570 nm. The % hemolysis was calculated as: H (%) = (OD_570nm_ sample−OD_570nm_ PBS)/(OD_570nm_ water−OD_570nm_ PBS)*100.

### Cell Viability Assay

The cell viability was analyzed by MTT assay. Briefly, BMDCs were cultured with gradient dilutions of each lipopeptide for 48 h. After that, 20 μL of a MTT solution (5 mg/ml) were added per well and incubated at 37°C for 4 h. Then, the mixture was removed from the well and 100 μL DMSO were added. The absorbance was detected at a wavelength of 570 nm. % surviving cells = Mean optical absorption of cells exposed to the lipopeptide/Mean optical absorption of control cells × 100.

### BMDCs Generation

Bone marrow-derived dendritic cells (BMDCs) were isolated using methods described previously (26, 27). Briefly, the cells were obtained by flushing the femurs of female BALB/C mice, then differentiated in RPMI 1640 medium containing 10% FBS and supplemented with IL-4 (10ng/mL) and GM-CSF (10ng/mL) for 7 days at 37°C in 5% CO_2_.

### FCM (Flow Cytometer)

BMDCs were collected after being treated with each lipopeptidet for 48h. The expressions of surface markers on BMDCs were detected by FCM with a FACS Canto II (BD Biosciences). The cells were stained with CD11c-FITC (Biolegend, 117306), CD86-PerCP (Biolegend, 105026), CD80-APC (Biolegend, 104714) and CD40-PE (Biolegend, 124610) at 4°C for 30min. After washing with PBS, the expression of costimulatory molecules was detected by flow cytometer.

### Cytokines Assay

BMDCs (4 × 10^6^ cells/well in 4mL RMPI-1640 medium) were stimulated with either synthetic lipopeptide (1 μg/mL) or PBS for 48 h. The expressions of IL-1β, TNF-α and IL-12 were analyzed by ELISA kit according to the manufacturer’s instructions.

### Animal Immunization

For OVA immunization, mice were divided into 7 groups randomly. Mice were the mice were anesthetized with pentobarbital sodium (50 mg/kg) and then intranasally immunized with PBS, OVA (10 μg), OVA (10 μg) plus one of the following lipopeptides: LP1-30, LP1-34, LP2-2 and LP2-3 or OVA plus Pam_2_CSK_4_ on day 0, 14, 28. OVA plus Pam_2_CSK_4_ group served as positive control. For SARS-CoV-2 recombinant RBD (rRBD) immunization, mice were intranasally immunized at two-week intervals with PBS, rRBD, rRBD plus LP1-34, rRBD plus LP2-2 or rRBD plus Pam_2_CSK_4_. Two weeks post the final immunization, mice were sacrificed and sera, saliva, stomach homogenates, intestinal lavage fluid and vaginal wash were collected for further analysis.

### ELISA

Specific IgG and sIgA levels were determined by standard indirect ELISA. 96-well flat bottom plates were coated with OVA or rRBD and incubated overnight at 4°C. After blocking with 5% BSA in PBST, the diluted samples were added into the plates and incubated at 37°C for 1h. HRP-conjugated goat anti-mouse IgG, IgG1, IgG2a or IgA was used as a secondary antibody. TMB substrate solution was added each well and incubated about 5-10 min. Then, the reaction was ended by adding stop solution. Finally, the optical density was detected by at 450nm in a micro-plate reader (Thermo Scientific).

### ELISpot

T cell responses were evaluated by ELISPOT assay using a mouse IFN-γ ELISPOT^PLUS^ kit (Mabtech) following the manufacturer’s instructions. Briefly, splenocytes from immunized mice were incubated for 48 hours in ELISpot plates in the absence or presence of 10 μg/mL of OVA_257–264_ peptide or OVA_323–339_ peptide. The number of specific IFN-γ-forming cells was calculated by subtracting the number spot-forming cells cultured with peptides from the number of spot-forming cells cultured without peptides.

### SARS-CoV-2 Neutralizing Antibody Detection (ELISA)

SARS-CoV-2 neutralizing antibody detection was performed according to the manufacturer’s instruction (zybio). Briefly, the diluted sera, BALF or nasal wash mixed with HRP-conjugated RBD were added into the hACE2-coated plate, and then the plate was incubated at 37°C for 1h. After washing the plate, the TMB substrate was added and incubated at 37°C for 10min. Finally, the reaction was ended by adding the stop solution and the absorbance was measured with a micro-plate reader (Thermo Scientific) at 450 nm.

### SARS-CoV-2 Pseudovirus Neutralization Assay

The SARS-CoV-2 pseudoviruses neutralization assay was completed in an approach similar to as described previously (28, 29). The day before the experiment, HEK293T-hACE2 cells were seeded in 96-well tissue culture plates at a density of 2 × 104 cells/well overnight. Vaccinated mouse sera serially diluted two-fold starting at a 1:50 dilution for assay. Serum samples were mixed with 2 µL of pseudovirus (Longzoe) for 30 minutes and added to HEK293T-hACE2 cells. After 48h, cells were lysed using Luciferase Assay (Promega) according to the manufacturer’s instructions and relative luminescence units (RLU) were measured by the Thermo Fisher plate reader. SARS-CoV-2 pseudovirus neutralization titers were defined as the sample dilution at which a 50% reduction in RLU was observed relative to the average of the virus control wells. The SARS-Cov-2 Pseudovirus Neutralization IC50 was then calculated using GraphPad Prism (version 8.4).

### Support Vector Machine (SVM)

SVM is used to find a separating hyperplane in the *k*-dimensional space under the given constraints and separating samples into two classes. The SVM solution to the optimal separating hyperplane can be expressed as:


$$\underset{w,b,\xi }{\text{min }}\frac{1}{2}║w{║}^{2}+C\overset{n}{\underset{i=1}{\sum }}\xi_{i}$$

s.t. *y_i_* (*w* ^T^ *Φ* (*x_i_*) + *b*) – (1 – *ξ_i_*) ≥ 0, *i* = 1,…, *n*.

**Table:** 

*k*: Number of features	*n*: Number of training vectors	*w:* Normal vector
*b*: Intercept term	*x_i_:* Training vector	*y_i_:* Classification label
*C*: Penalty parameter	*ξ_i_*: Training error	*Φ*(*x*): kernel function

After solving the hyperplane *w* ^T^ *x*+*b*=0, the larger the *w_i_*, the greater the contribution of the *i*-th feature to the hyperplane, which suggests the *i*-th feature of the sequence is more significant to the effect of lipopeptides. In this study, all the 9 features were retrieved from the ProtParam tool on Expasy (https://web.expasy.org/protparam/), as shown in **Table 1**. For the vector *y*= (*y*_1_; …; *y_n_*), we defined *y_i_* = 1 if the lipopeptide *x_i_* had the agonistic activity, and *y_i_* = −1 if not (compared to the agonistic activity of Pam_2_CSK_4_). All the SVM calculations were performed in libsvm 3.18 (30), a general library for support vector classification and regression. Before calculating, all the values of 9 features were uniformized into [-1,1] by the svm-scale tool in libsvm. The radial basis function given by exp(-*γ*||*x_i_*-*x_j_* ||^2^) was used as the kernel function *Φ*(*x*) for all our calculations. The penalty parameter *C* and the kernel parameter *γ* were determined by the grid-search tool in libsvm.

**Table 1:** The features corresponded to every *w*_i_.

*w*_i_	Features
***w*_1_**	The number of amino acids
***w*_2_**	Molecular weight
***w*_3_**	Theoretical isoelectric point
***w*_4_**	Total number of negatively charged residues (Asp + Glu):
***w*_5_**	Total number of positively charged residues (Arg + Lys):
***w*_6_**	Extinction coefficient
***w*_7_**	Instability index
***w*_8_**	Aliphatic index
***w*_9_**	Grand average of hydropathicity (GRAVY)

### Statistical Analysis

All statistical analysis were performed by GraphPad Prism 7.0 software. The difference between two groups was analyzed by t-test. Multiple comparisons were analyzed by one-way ANOVA. *P* < 0.05 were considered statistically significant. All data were shown as a mean ± standard deviation (SD).

## Results

### The Design and Synthesis of the Novel Lipopeptides

**Figure 1:**
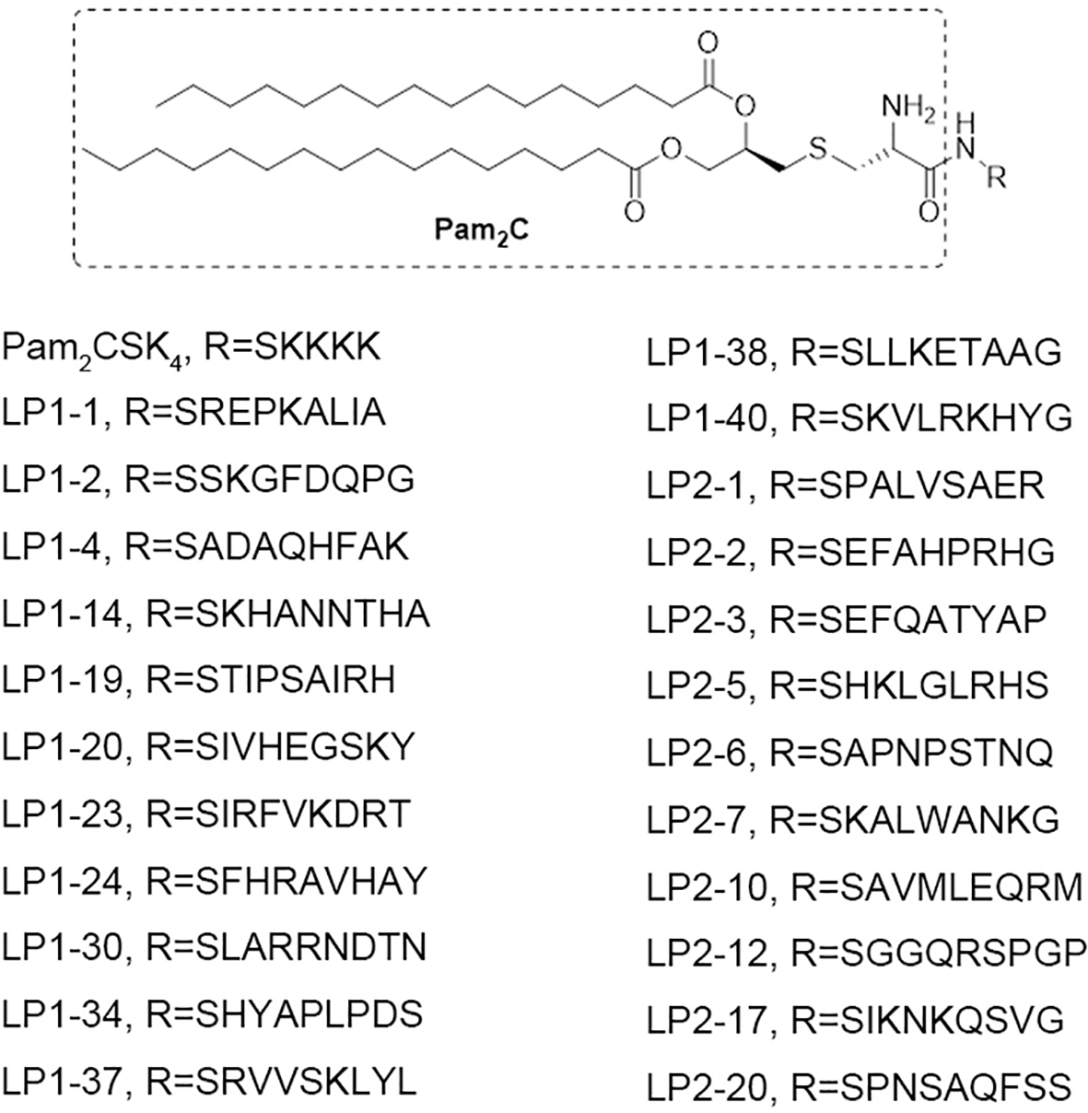
The chemical structures of the 23 novel synthetic lipopeptides.

To explore the influence of peptide sequence in the lipopeptide on its TLR2 activity, 100 peptide sequences were randomly generated using online Sequence Manipulation Suite (http://bioinformatics.org/sms2/sample_protein.html) based on amino acid composition (%) in the UniProtKB/Swiss-Prot data bank (**Table S1**). We tried to synthesize the 100 lipopeptides containing the above generated sequences, however only 23 of them were successfully synthesized with the purity of more than 95% (**Figure 1**).

### Identification of Novel Lipopeptides Which Could Activate TLR2 Signaling

**Figure 2:**
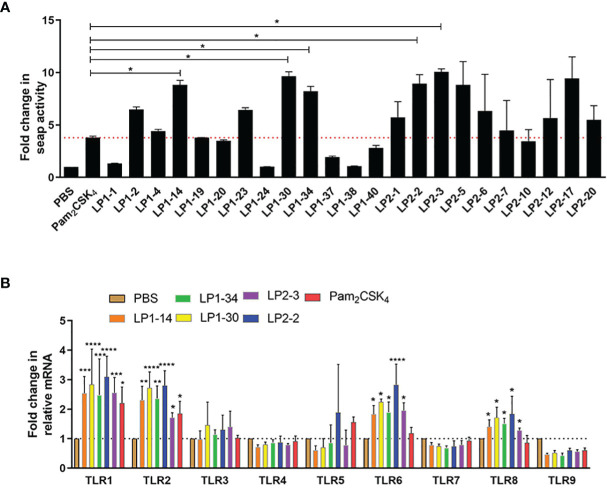
TLR2 agonistic activity of the novel lipopeptides. **(A)** SEAP activity was measured in HEK-Blue™ mTLR2 cells treated with synthetic lipopeptides (n=3). **(B)** The gene expressions of TLR1-TLR9 were detected by real-time RT-PCR (n=3). Data were presented as the mean ± SD. *p < 0.05, **p < 0.01, ***p < 0.001, ****p < 0.0001, compared with PBS control.

To identify the lipopeptides which could activate TLR2 signaling more effectively compared with Pam_2_CSK_4_, SEAP activity was measured after HEK-Blue™ mTLR2 cells treated with each novel lipopeptide. As shown in **Figure 2A**, LP1-14, LP1-30, LP1-34, LP2-2 and LP2-3 treatment showed more increased SEAP activity than Pam_2_CSK_4_, indicating that they may be able to induce more robust TLR2 signaling. The gene expression of TLR2 was also significantly increased in the presence of the above 5 novel lipopeptides. In addition, the effect of those lipopeptides on other TLR signaling were measured, as shown in **Figure 2B**, they were also capable of significantly triggering TLR1, TLR6 and TLR8 signaling.

### *In Vitro* Safety of the Novel Lipopeptides

The cytotoxicity of the 5 novel synthetic lipopeptides on BMDCs was evaluated using MTT colorimetric assay. No significant toxicity was detected at test concentrations (10-200μg/mL), except that LP1-14 was slightly cytotoxic on BMDCs (**Figure S3A**). Moreover, compared with Pam_2_CSK_4_, the 5 synthetic lipopeptides induced less hemolysis after exposure in mice red blood cells (**Figure S3B**). The above results indicated that those novel synthetic lipopeptides except LP1-14 were safe *in vitro* and selected for the further study.

### Novel Lipopeptides Promoted BMDCs Maturation

**Figure 3:**
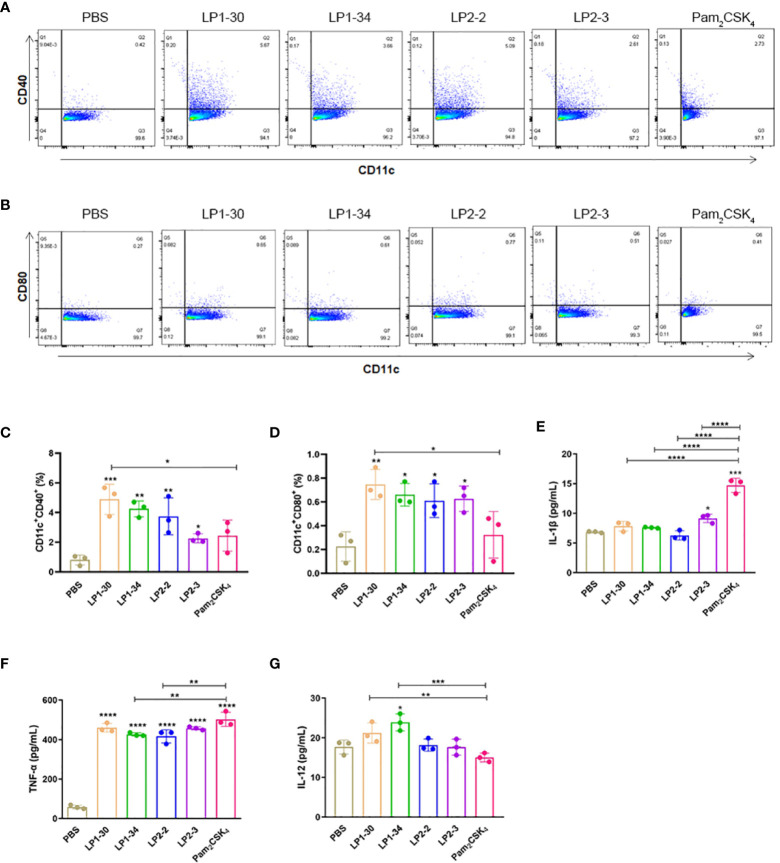
The expression of maturation surface markers and cytokines in the supernatants of BMDCs stimulated with the novel lipopeptides. BMDCs were treated with the synthetic lipopeptides respectively, and then cells were collected after 48h. The expression levels of CD40 and CD80 were analyzed by flow cytometry. **(A, B)** Representative flow cytometry dot plots. **(C, D)** Normalized expression level of maturation markers. **(E–G)** The expression of IL-1β, TNF-α and IL-12 in the supernatants of BMDCs were detected by ELISA (n=3). Data were presented as the mean ± SD. *p < 0.05, **p < 0.01, ***p < 0.001, ****p < 0.0001.

BMDCs were stimulated with PBS, LP1-30, LP1-34, LP2-2 and LP2-3 for 2 days, and the levels of the costimulatory molecules (CD40 and CD80) expression were evaluated by FACS. As shown in **Figures 3A–D**, those novel lipopeptides induce significantly increased CD80/CD86 expression on BMDCs, and LP1-30 showed more potent stimulating effect compared with Pam_2_CSK_4_, suggesting that the novel lipopeptides markedly induced maturation of BMDCs. In addition, secreted levels of cytokines from BMDCs were quantified by ELISA. The four lipopeptides increased production of pro-inflammatory cytokine TNF-α, but not IL-1β (**Figures 3E, F**). Compared with Pam_2_CSK_4,_ LP1-30 and LP1-34 treatment induced higher levels of IL-12 (**Figure 3G**), indicating that the novel lipopeptides had the potential to activate the innate immune response and T-cell mediated response.

### Novel Lipopeptides Enhanced Serum OVA-Specific IgG and IgA Response

**Figure 4:**
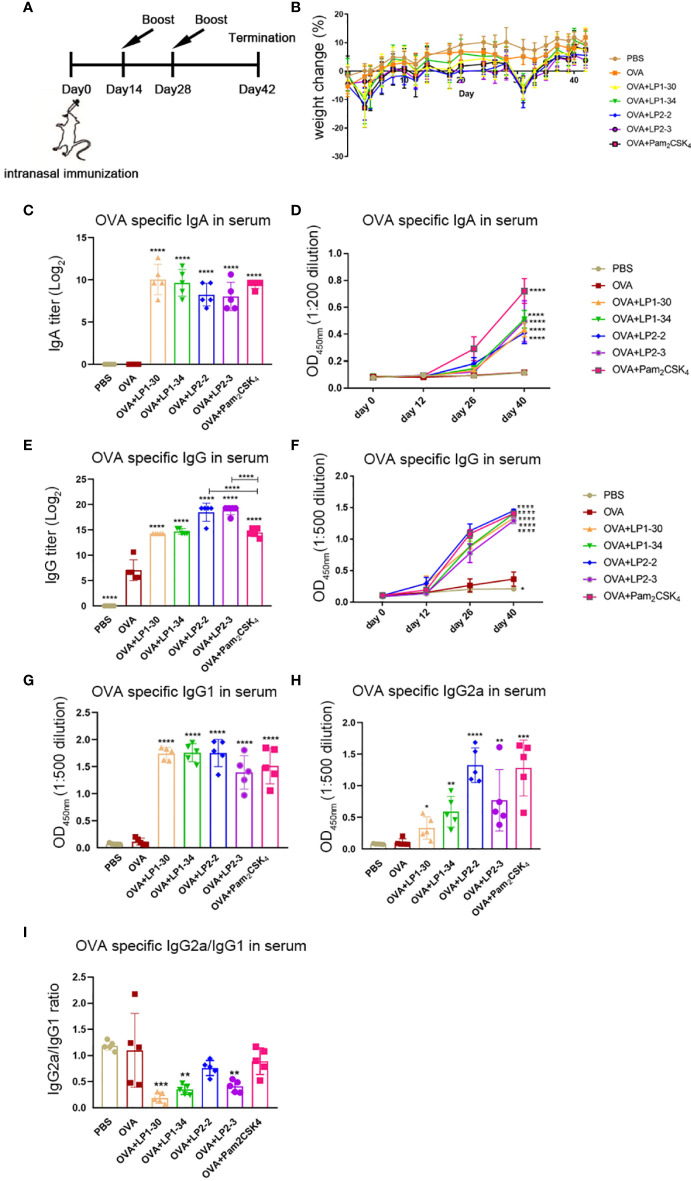
Serum specific IgG and IgA levels of mice immunized with OVA plus the novel lipopeptide. Female BALB/c mice were intranasally immunized with OVA plus LP1-14, LP1-30, LP1-34 or LP2-2 at day 0, 14 and 28. After 14 days of final immunization, mice were sacrificed. OVA plus Pam_2_CSK_4_ group served as positive control. **(A)** Timeline representation of immunization schedule and experimental procedures. **(B)** The body weight was recorded (n=5). The level of OVA specific IgA**(C, D)**, IgG **(E, F)**, IgG1 **(G)**, IgG2a **(H)** and IgG2a/IgG1 ratio **(I)** in sera were determined by ELISA (n=5). Data were presented as the mean ± SD. **p<0.01, **** *p*<0.0001, compared with OVA alone group.

In order to evaluate the mucosal adjuvant effect of the novel lipopeptides, mice were intranasally immunized with OVA plus LP1-30, LP1-34, LP2-2 or LP2-3, respectively (**Figure 4A**). There were no significant differences in body weight changes between the groups adjuvanted with each novel lipopeptide and Pam_2_CSK_4_ (**Figure 4B**). ELISA data showed that OVA plus LP1-30, LP1-34, LP2-2 or LP2-3 immunization significantly increased the level of serum specific IgG and IgA (**Figures 4C–F**). Moreover, LP2-2 and LP2-3 elicited more robust specific IgG response compared with Pam_2_CSK_4_. The levels of LP-specific antibody in the sera were also measured by ELISA. Though low levels of anti-LP1-30 IgG antibodies were detected in the OVA+LP1-30 group, this trend did not reach statistical significance. Other lipopeptides also failed to elicit specific IgG or IgA antibodies (**Figure S4**), suggesting that those might have no or few reactogenicity or immunogenecity. The IgG2a and IgG1 isotypes as markers of Th1 and Th2 lymphocytes respectively, OVA-specific IgG1 and IgG2a levels were also determined. As shown in **Figures 4G, H**, all synthetic lipopeptides significantly enhanced specific IgG1 response, and LP2-2, LP2-3, Pam_2_CSK_4_ also elevated the levels of specific IgG2a. Lower IgG2a/IgG1 ratio were observed when a lipopeptide used as the mucosal adjuvant, suggesting that those novel lipopeptides were more potent to drive Th2-biased immune response (**Figure 4I**).

### Novel Lipopeptides Elevated the Levels of OVA-Specific sIgA and IgG at Mucosal Sites

**Figure 5:**
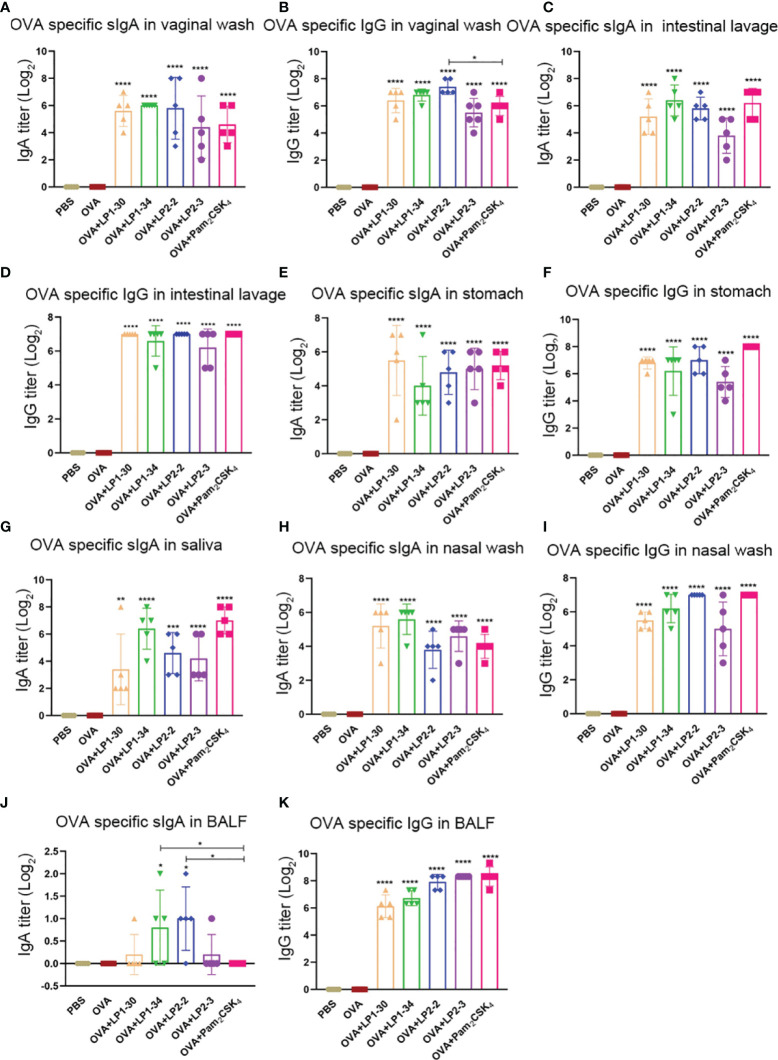
Mucosal specific sIgA and IgG profiles of mice immunized with OVA plus the novel lipopeptide. Female BALB/C mice were intranasally immunized with OVA plus LP1-14, LP1-30, LP1-34 or LP2-2 at day 0, 14 and 28. After 14 days of final immunization, mucosal fluids were collected. OVA plus Pam_2_CSK_4_ group served as positive control. **(A–E)** The OVA specific IgG in vaginal wash, intestinal lavage fluid, stomach homogenates, nasal wash and BALF were analyzed by ELISA (n=5) and the titer was calculated respectively. **(F–K)** The OVA specific IgG in vaginal wash, saliva, intestinal lavage fluid, stomach homogenates, nasal wash and BALF were analyzed by ELISA (n=5) and the titer was calculated respectively. Data were presented as the mean ± SD. *p < 0.05, **p < 0.01, ***p < 0.001, ****p < 0.0001, compared with OVA alone group.

The levels of specific sIgA and IgG at different mucosal sites were also determined. OVA plus the synthetic lipopeptides induced significantly higher levels of vaginal specific sIgA and IgG compared with OVA alone group (**Figure 5A**). LP1-30, LP1-34 and LP2-2 increased higher level of vaginal IgG compared with Pam_2_CSK_4_, but only adjuvanted with LP2-2 reached significance (**Figure 5B**). Besides, novel lipopeptides elevated the levels of OVA-specific sIgA and IgG in the intestinal lavage fluid, stomach homogenates and saliva (**Figures 5C–G**). Importantly, immunization with OVA plus the novel lipopeptides enhanced specific sIgA and IgG response in the nasal mucosa (**Figures 5H, I**). Moreover, LP1-34 and LP2-2 assisted OVA to induce higher level of sIgA in BALF compared with Pam_2_CSK_4_ (**Figures 5J, K**).

### Adjuvanted With Novel Lipopeptides Failed to Elicit Substantial OVA-Specific T Cell Response

**Figure 6:**
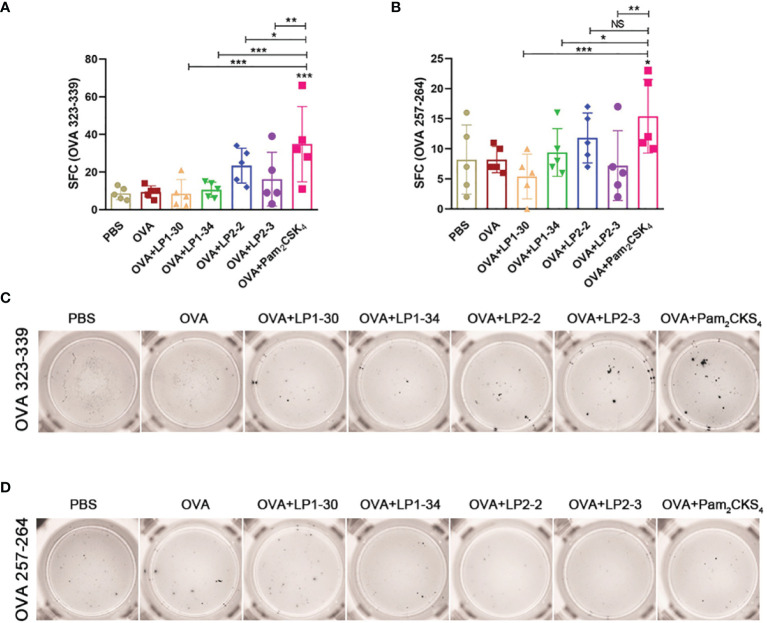
Specific T cell response elicited by OVA plus the novel lipopeptide. Female BALB/C mice were intranasally immunized with OVA plus LP1-14, LP1-30, LP1-34 or LP2-2 at day 0, 14 and 28. After 14 days of final immunization, mice splenic lymphocytes were stimulated by OVA_257-264_ peptide or OVA_323-339_ peptide respectively. OVA plus Pam_2_CSK_4_ group served as positive control. **(A, B)** Numbers of IFN-γ spot forming cells in splenic lymphocytes were detected by ELISpot (n=5). **(C, D)** Representative well images of ELISpot assay. Data were presented as the mean ± SD. ns, no significant, *p < 0.05, **p < 0.01, ***p < 0.001.

To further investigate whether novel lipopeptides enhanced specific cellular immune responses, ELISpot assay was performed on splenic lymphocytes collected from immunized mice for detecting IFN-γ-forming OVA_257-264_-specific CD8^+^ T cells and OVA_323-339_-specific CD4^+^ T cells. As shown in **Figures 6A–D**, adjuvanted with the novel synthetic lipopeptides failed to elicit substantial specific T cell response, while Pam_2_CSK_4_ slightly elevated both CD4^+^ and CD8^+^ T cell response.

### Novel Lipopeptides Enhanced Respiratory Mucosal RBD-Specific sIgA and IgG Response

**Figure 7:**
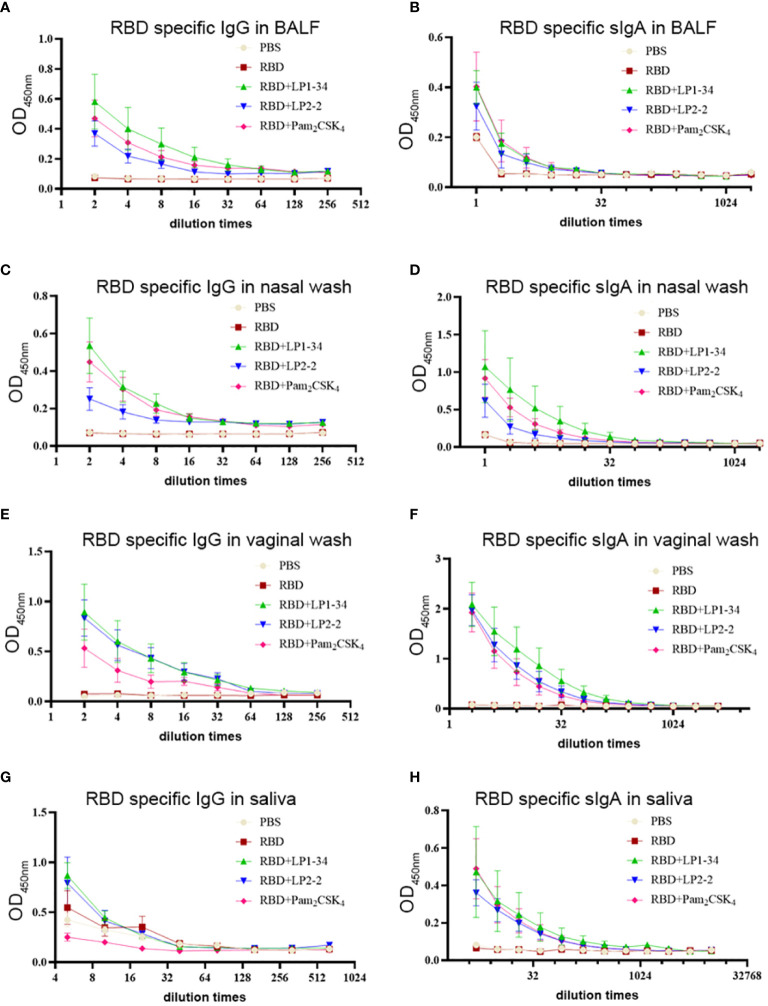
RBD-specific sIgA and IgG response at mucosal sites elicited by rRBD plus synthetic lipopeptides. Female BALB/C mice were intranasally immunized with rRBD plus LP1-34, LP2-2 or Pam_2_CSK_4_ 0, 14 and 28. After 14 days of final immunization, mucosal fluids were collected for ELISA assay. **(A, B)** RBD specific IgG and IgA in BALF were detected by ELISA (n=5). **(C, D)** RBD specific IgG and IgA in nasal wash were measured by ELISA (n=5). **(E, F)** RBD specific IgG and IgA in vaginal wash were detected by ELISA (n=5). **(G, H)** RBD specific IgG and IgA in saliva were detected by ELISA (n=5). Data were presented as the mean ± SD.

As SARS-CoV-2 initially infects the respiratory tract, vaccine-mediated respiratory mucosal antibodies and systemic IgG are critical for the protection against the pathogen. The possibility of the novel synthetic lipopeptides as the mucosal adjuvant for the SARS-CoV-2 rRBD was investigated. RBD-specific IgG or sIgA in BALF and nasal wash were not observed in the mice intranasally immunized with rRBD alone. However, in combination with LP1-34 or LP2-2, rRBD elicited significant mucosal sIgA and IgG response in the respiratory tract (**Figures 7A–D**). In addition, synthetic lipopeptides significantly elevated salivary and vaginal specific sIgA and IgG levels (**Figures 7E–H**). Generally, the ability of the LP1-34 to enhance the level of mucosal sIgA and IgG was stronger than that of Pam_2_CSK_4_.

### rRBD Plus Synthetic Lipopeptides Induced Significant Serum Specific IgG and IgA Response

**Figure 8:**
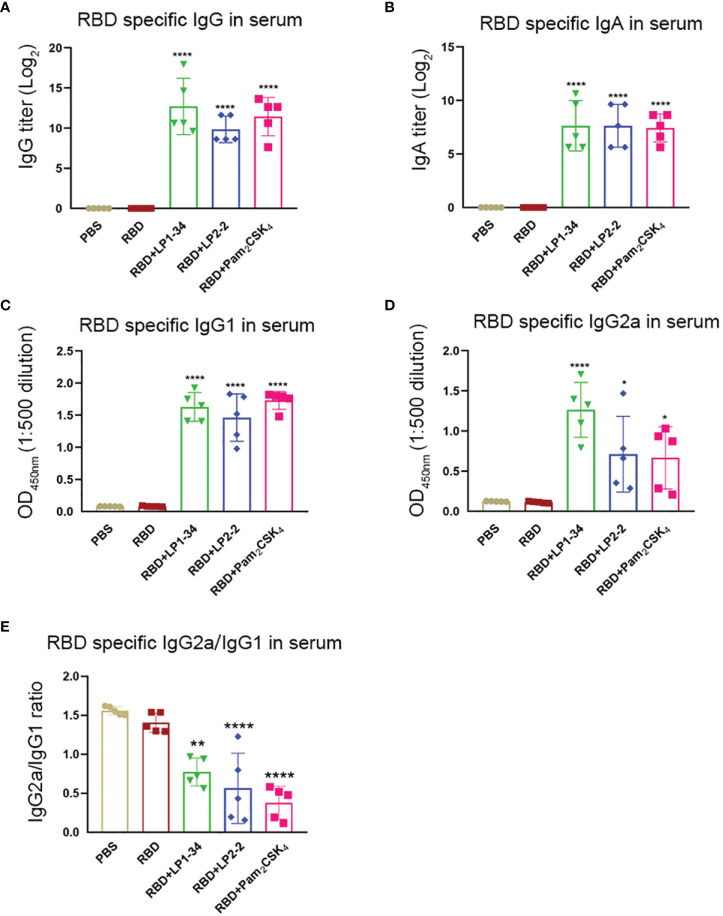
Serum RBD-specific IgG, IgG subclass and IgA profiles of mice immunized with rRBD plus novel lipopeptides. Female BALB/C mice were intranasally immunized with rRBD plus LP1-34, LP2-2 or Pam_2_CSK_4_ at day 0, 14 and 28. After 14 days of final immunization, sera were collected from immunized mice. The RBD specific levels of IgG **(A)**, IgA **(B)**, IgG1**(C)**, IgG2a **(D)** and IgG2a/IgG1 ratio **(E)** in serum were determined by ELISA (n=5). Data were presented as the mean ± SD. *p < 0.05, **p < 0.01, ****p < 0.0001, compared with rRBD alone control.

Similar to the results of mucosal antibodies, intranasal immunization with rRBD alone failed to induce significant specific serum IgG and IgA response, possibly due to its low immunogenicity (**Figures 8A, B**). However, using LP1-34, LP2-2 or Pam_2_CSK_4_ as the adjuvant significantly improved the levels of RBD-specific serum IgG and IgA. In addition, both specific IgG2a and IgG1 levels were elevated by LP1-34, LP2-2 or Pam_2_CSK_4_ compared with rRBD alone group, and LP1-34 induced the highest specific IgG2a response (**Figures 8C, D**). IgG2a/IgG1 ratio showed that rRBD plus the lipopeptide induced a Th2-biased response (**Figure 8E**).

### Novel Lipopeptides Significantly Increased Levels of rRBD-Induced SARS-CoV-2 Neutralizing Antibody in Sera, BALF and Nasal Wash

**Figure 9:**
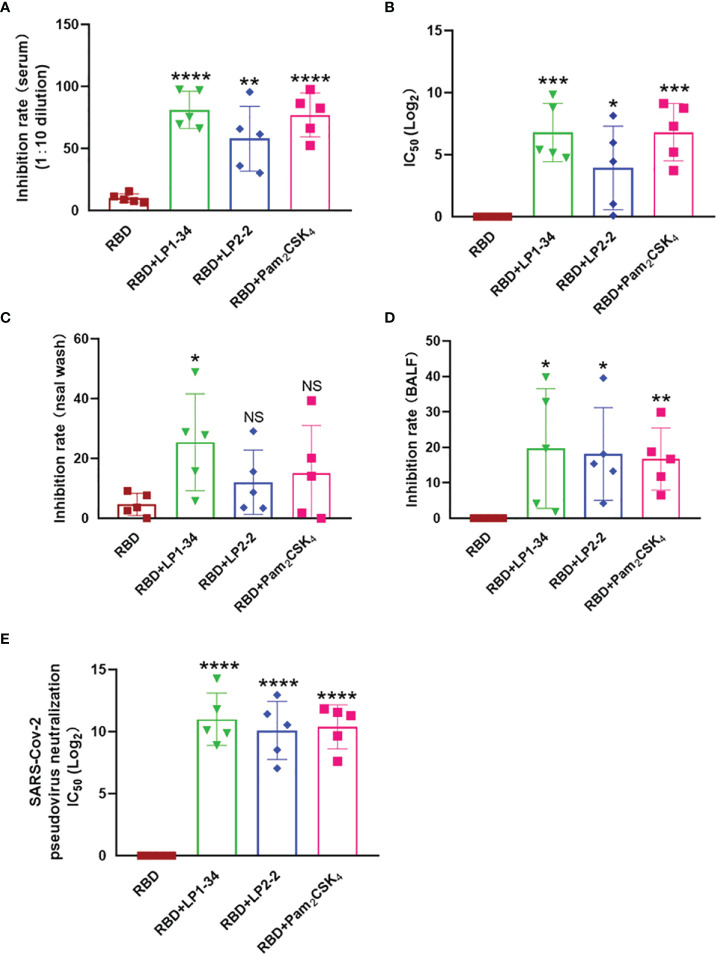
SARS-CoV-2 neutralizing antibody in sera, BALF and nasal wash. Female BALB/C mice were intranasally immunized with rRBD plus LP1-34, LP2-2 or Pam_2_CSK_4_. After 14 days of final immunization, sera, BALF and nasal wash were collected. **(A, B)** The serum inhibition rate and IC_50_ of RBD-ACE2 interactions analyzed by ELISA (n=5). **(C, D)** The inhibition rate of RBD-ACE2 interactions by nasal wash and BALF were analyzed by ELISA (n=5). SARS-CoV-2 pseudoviruses neutralization IC50 in serum was determined **(E)**. Data were presented as the mean ± SD. ns, no significant, *p < 0.05, **p < 0.01, ***p < 0.001, ****p < 0.0001, compared with rRBD alone group.

Because viral infection is initiated by binding Spike RBD to the human ACE2, vaccine-induced antibody that block the RBD-ACE2 interaction is thought to play a major role in protection against SARS-CoV-2 infection. The levels of neutralizing antibodies that block RBD-ACE2 interactions were determined. LP1-34, LP2-2 and Pam_2_CSK_4_ significantly elevated levels of rRBD-induced SARS-CoV-2 neutralizing antibody in sera (**Figures 9A, B**). In addition, the novel lipopeptides, especially LP1-34, improved the ability of BALF and nasal wash to inhibit RBD interaction with ACE2 (**Figures 9C, D**), indicating that the novel lipopeptides have the potential to enhance antigen-mediated prevention of the viral entry at respiratory mucosal sites. More importantly, LP1-34, LP2-2 and Pam2CSK4 could significantly augment the elicitation of SARS-CoV-2 pseudovirus neutralizing antibody titers by rRBD immunogen (**Figure 9E**).

### The Relationship Between Peptide Sequence in Lipopeptides and TLR2 Activity Was Analyzed by SVM

SVM was used to investigate the relationship between the peptide sequence in the lipopeptide and its TLR2 activity. As shown in **Tables 1** and **2**, three features with a strong positive contribution were *w*_3_ (the number of Arg + Lys), *w*_4_ (the number of Asp + Glu) and *w*_5_ (theoretical isoelectric point), which suggested that charged residues might be beneficial to the TLR2 agonistic activity. Aliphatic index and GRAVY showed the negative contribution. The GRAVY value for a peptide was calculated as the sum of hydropathy values of all the amino acids, divided by the number of residues in the sequence (31). The aliphatic index of a peptide was defined as the relative volume occupied by aliphatic side chains (alanine, valine, isoleucine, and leucine) (32). Hydropathy was represented as negative values in both features. Thus, these two features showed similar result that lipophilic residues might adversely affect the TLR2 activity. Furthermore, the accuracy rate of prediction was 93.10% for the training samples.

**Table 2:** All the weight coefficients *w_i_* s and the contribution of corresponded features to the TLR2 activity.

w_i_s	Features of the peptide in the lipopeptide	Coefficient Value	Contribution
***w*_1_**	The number of amino acids	0.0001	0
***w*_2_**	Molecular weight	-0.0410	0
***w*_3_**	Theoretical isoelectric point	0.7410	++
***w*_4_**	Total number of negatively charged residues	0.8244	++
***w*_5_**	Total number of positively charged residues	0.8210	++
***w*_6_**	Extinction coefficient	-0.0008	0
***w*_7_**	Instability index	0.0287	0
***w*_8_**	Aliphatic index	-0.1638	–
***w*_9_**	Grand average of hydropathicity (GRAVY)	-0.3258	–

+: positive contribution.

-: negative contribution.

0: nearly no contribution.

## Discussion

Toll-like receptors (TLRs) agonists used in combination with antigens are showing initial success in terms of enhancing immune responses. TLR4 ligand MPL and TLR9 ligand CpG ODN have been included in vaccines that are licensed (33, 34), implicating them as safe and efficacious adjuvants.

Pam_2_CSK_4_, as one of the most potent dual (human and murine) TLR2 agonist, is a potent adjuvant which can enhance specific immune response used in combination with an antigen (35). In order to discover novel lipopeptides those are more potent in activating TLR2 than Pam_2_CSK_4_, 100 lipopeptides were designed, 23 of which were successfully synthesized in the current study. TLR2 activity test results showed that LP1-14, LP1-30, LP1-34 and LP2-2 possessed more powerful TLR2 agonistic activity compared with Pam_2_CSK_4_. In addition, the synthetic lipopeptides also enhanced TLR1 expression, and TLR1 activation may also contribute to their adjuvant effect. To discover how the peptide sequence of lipopeptides affects their TLR2 activity, SVM was used to recognize the patterns between the features of lipopeptides and their agonistic activity. We found that charged residues of the lipopeptide might be beneficial to the agonist activity, while lipophilic residues have negative effect on the agonist activity.

A number of studies have shown that lipopeptides have a significant mucosal adjuvant effect (36–38). MALP-2, the mycoplasma-derived lipopeptide, elevated the level of sIgA in BALF and vaginal lavages when intranasal co-administration with a soluble antigen (25). Intranasally immunization with inactivated EV71 vaccine plus FSL-1 (a synthetic diacylated lipopeptide recognized with TLR2/6) also resulted in higher level of EV71-specific IgG and IgA in serum, nasal washes, BALF, and feces compared with the control group (37). Our previous study found that lipopeptides, which mimic the terminal structure of the native HpaA (*H. pylori* adhesin A), improved the protective effect of rHpaA against *H. pylori* infection (21). In this study, the mucosal adjuvant effects of the novel synthetic lipopeptides were also systematically investigated. LP1-30, LP1-34, LP2-2 and LP2-3 enhanced the production of IgG and IgA in serum, saliva, nasal wash, BALF, intestinal lavage fluid, stomach homogenate and vaginal wash compared with the non-adjuvant group. In addition, adjuvanted with the novel lipopeptides resulted in lower serum IgG2a/IgG1 ratio, suggesting that those novel lipopeptides were more potent to drive Th2-biased immune response. Among the four novel lipopeptides, LP1-34 and LP2-2 assisted OVA to induce more profound specific IgG in sera or sIgA in BALF compared with Pam_2_CSK_4_.

Since the outbreak of COVID-19, the spread of SARS-CoV-2 has impacted nearly every aspect of society worldwide. Although a variety of effective COVID-19 vaccines have been implemented (39–41), most studies have focused exclusively on the evaluation of vaccine-mediated serum IgG and systemic T cell response. As SARS-CoV-2 initially infects respiratory tract, mucosal response induced by the vaccine may be crucial in providing first line defense at the site of viral entry (42). Intranasal immunization is a common way to induce respiratory mucosal response. Though the SARS-CoV-2 receptor-binding domain (RBD) is an attractive vaccine target, its immunogenicity is limited due to low molecular weight. Adjuvants had been used to enhance the immunogenicity of rRBD and result in increased mucosal sIgA titers (43). In our study, intranasally immunization with rRBD plus either LP1-34, LP2-2 or Pam_2_CSK_4_ elicited a robust systemic humoral immunity with higher titers of serum IgG antibodies as well as a significant mucosal immunity with enhanced levels of IgG and sIgA in nasal wash, BALF, saliva and vaginal wash. Moreover, sera from mice immunized plus each novel lipopeptides showed enhanced inhibition of the RBD-ACE2 interaction. Importantly, LP1-34 significantly improved the ability of BALF and nasal wash to inhibit RBD interaction with ACE2.

In conclusion, we synthesized 23 novel lipopeptides, and 5 of them showed more potent TLR2 agonistic activity *in vitro* compared with Pam_2_CSK_4_. Based on the results of safety test, LP1-14, LP1-30, LP1-34 and LP2-2 were selected for the further evaluation of mucosal adjuvant activity *in vivo*. In combination with OVA, LP1-34 and LP2-2 showed more potent mucosal adjuvant effect than Pam_2_CSK_4_. In addition, the novel lipopeptides, especially LP2-2, enhanced systemic and mucosal antibody response when intranasally administrated with rRBD, suggesting that lipopeptides may serve as a potent adjuvant for COVID-19 vaccine. In this study, our findings provided clues to the relationship between the peptide sequence in the lipopeptide affect its TLR2 activity, which lays a foundation for the rational design of lipopeptide adjuvants in the future.

## Data Availability Statement

The original contributions presented in the study are included in the article/**Supplementary Material**. Further inquiries can be directed to the corresponding authors.

## Ethics Statement

The animal study was reviewed and approved by Animal Ethical and Experimental Committee of the Third Military Medical University.

## Author Contributions

LM, CL, and J-YL contributed equally to this work. H-BL and Q-MZ designed experiments. LM, CL, J-YL, ZJ, R-YX, RF, G-CL, YD, and HC carried out experiments. LM, Z-LJ, CL, and J-YL analyzed experimental results. H-BL and Q-MZ wrote the manuscript. All authors contributed to the article and approved the submitted version.

## Funding

This work was supported by Chinese National Natural Science Foundation Project (No. 31670936, 82041045) and Key Program of Chongqing Natural Science Foundation (No. cstc2020jcyj-zdxmX0027).

## Conflict of Interest

The authors declare that the research was conducted in the absence of any commercial or financial relationships that could be construed as a potential conflict of interest.

## Publisher’s Note

All claims expressed in this article are solely those of the authors and do not necessarily represent those of their affiliated organizations, or those of the publisher, the editors and the reviewers. Any product that may be evaluated in this article, or claim that may be made by its manufacturer, is not guaranteed or endorsed by the publisher.
