# Supplementary material for: Novel Synthetic Lipopeptides as Potential Mucosal Adjuvants Enhanced SARS-CoV-2 rRBD-Induced Immune Response
Source: Front Immunol. 2022 Mar 9;13:833418. doi: 10.3389/fimmu.2022.833418 (PMC8959576; doi:10.3389/fimmu.2022.833418)
Supplement: Supplementary file 1 [file DataSheet_1.docx]

**Supplementary information**

**Novel synthetic lipopeptides as potential mucosal adjuvants enhanced SARS-CoV-2 rRBD-induced immune response**

Ling Mao^1#^, Chang Liu^1#^, Jing-Yi Liu^1#^, Zi-Li Jin^1^, Zhe Jin^1^, Ruo-Yi Xue^1^, Rang Feng^1^, Guo-Cheng Li^1^, Yan Deng^1^, Hao Cheng^1^, Quan-Ming Zou^1^*, Hai-Bo Li^1^*

^1^ National Engineering Research Center of Immunological Products, Department of Microbiology and Biochemical Pharmacy, College of Pharmacy, Third Military Medical University, Chongqing 400038, P.R. China.

^#^ Authors contributed equally

Correspondence to Prof. Hai-Bo Li,

College of Pharmacy, Third Military Medical University, Chongqing 400038, P.R. China

E-mail address: lihaibo@tmmu.edu.cn (H-B. Li)

Or Prof. Quan-Ming Zou,

College of Pharmacy, Third Military Medical University, Chongqing 400038, P.R. China

E-mail address: qmzou2007@163.com (Q-M. Zou)

**1. Supplementary Methods**

**Chemical Synthesis**

**General.** Unless otherwise noted, materials were purchased from commercial suppliers and were used as received. Solvents used for chromatography were distilled prior to use. All anhydrous reactions were carried out under a nitrogen atmosphere using oven-dried glassware. ^1^H nuclear magnetic resonance (NMR) spectra were recorded on a BRUKER 400 MHz NMR spectrometer. ^1^H NMR spectra are reported in parts per million (ppm) downfield from an internal standard, tetramethylsilane (0 ppm). The purity of the lipopeptides was analyzed by HPLC equipped with a C18 column (SinoChrom ODS-BP, 4.6×250 mm, 5 μm). The mobile phase consisting of solvent A (0.1% TFA in 100% water) and solvent B (0.1% TFA in 100% acetonitrile) was used as the eluent. The structures of the lipopeptides were characterized by ESI-MS (Agilent-6125B).

**Synthesis of L-cystine bis-t-butyl ester (2)**

L-cystine (**1**, 20.8 mL, 5 g) was dissolved in 70% HClO_4_（8.4 mL）, then *tert*-butyl acetate（50 mL, 374.4 mmol）was added dropwise cold with an ice-bath. The resulting solution was stirred at RT for 2 days. The reaction mixture was ice-cooled and the pH was adjusted to 11 using 4N aq NaOH(32 g NaOH in 200 mL H_2_O). The temperature of the reaction was brought back to RT, extracted with EA 6 times and the resulting organic phases were combined together, dried over Na_2_SO_4_, and concentrated in vacuo to give **2** (4.5g, 62% yield) as yellow oil.

**Synthesis of N^α^,N^α^-Bis-Fmoc-L-cystine bis(*tert*-butyl ester) (3)**

To a solution of **2** (1.6 g, 4.54 mmol) in 50mL THF was added Fmoc-OSu (1.53 g×2, 4.54 mmol×2).The mixture was ice-cooled. N-Methylmorpholine (NMM) (1 mL, 9.08 mmol) was added dropwise and continued to stir overnight. To this reaction mixture, ethyl acetate (60 ml) and 10% citric acid solution (25 mL) were added to thus separate the mixture into different phase to obtain an organic phase. The resulting organic phase was further washed twice with 10% citric acid solution (25mL), brine, and then concentrated to give a slurry-like residue, purified by silica gel column chromatography to give 3 (2.9g，80% yield) as yellow solid.

**Synthesis of (R)-Glycidol (5)**

To a solution of (R)-3-Chloro-1,2-propanediol (**4**, 2 g, 18.2 mmol) in DCM 25 ml was added K_2_CO_3_ (6.3 g, 45.45 mmol). The resulting mixture was vacuum filtered through celite and concentrated in vacuum to give crude **5** (1.13g, 84% yield) as colorless oil, which was used without further purification.

**Synthesis of N-(((9H-fluoren-9-yl)methoxy)carbonyl)-S-((R)-2,3-dihydroxypropyl)-L- cysteine *tert*-butyl ester (6)**

Zn (153 mg, 2.35 mmol) and a mixture of MeOH, conc. HCl and conc. H_2_SO_4_ (100:7:1, 18 mL) were added to an ice-cooled solution of **3** (500 mg, 0.63 mmol) in DCM (3.6 mL). After stirring for 30 mins at 0 ℃, **5** (466mg,6.3mmol) was added to the reaction mixture and stirred for 3 hours at 40 ℃. The mixture was concentrated to a small volume (half) and preapitates were removed by filtration. Saturated NaCl were added to filtrate and extracted with DCM twice. The combine organic layer was dried with Na_2_SO_4_ and concentrated. The oily residue was purified by CC to give **6** (1.01 g, 59% yield) as oil.

**Synthesis of Fmoc-Pam_2_Cys *tert*-butyl ester (7)**

To a solution of palmitic acid (108 mg, 0.42 mmol) in DMF (1 mL) were added **6** (100 mg, 0.21 mmol) and DMAP (5 mg, 0.042 mmol), after cooling with ice, EDCI (8 mg, 0.042 mmol) was added, and stirred for 6h with gradually warming up to RT. Water was added to the reaction mixture, extracted with DCM, and purified by CC to give **7** (35 mg, 17.6% yield).

**Synthesis of Fmoc-Pam_2_Cys**

To a solution of **7** (700 mg, 0.737 mmol) in DCM 5mL was added 2mL TFA, and the mixture was stirred at RT for 2h. Purification by recrystallization from (PE/EA) gave Fmoc-Pam_2_Cys (183.7 mg, 27.9% yield) as white solid. ^1^H NMR (400 MHz, CDCl_3_) *δ* 7.78 (d, *J* = 4.0 Hz, 2H), 7.62 (d, *J* = 4.0 Hz, 2H), 7.41 (t, *J* = 8.0 Hz, 2H), 7.35 (t, *J* = 8.0 Hz, 2H), 5.81 (d, *J* = 4.0 Hz, 1H), 5.20 (s, 1H), 4.69 (d, *J* = 4.0 Hz, 1H), 4.44 – 4.40 (m, 3H), 4.37 (t, *J* = 4.0 Hz, 1H), 4.28-4.26 (m, 1H), 3.20 – 3.08 (m, 2H), 2.80 – 2.79 (m, 2H), 2.36 – 2.30 (m, 4H), 1.63 – 1.62 (m, 4H), 1.28 – 1.26 (m, 48H), 0.92 – 0.89 (m, 6H). LC-MS (ESI+): m/z 893.2 (M-H)+; [α]^23^_D_= 6.57°（C=1,CHCl_3_）.

**2. Supplementary figures**

**Fig. S1** 1H NMR spectrum (CDCl_3_) of Fmoc-Pam_2_Cys

**
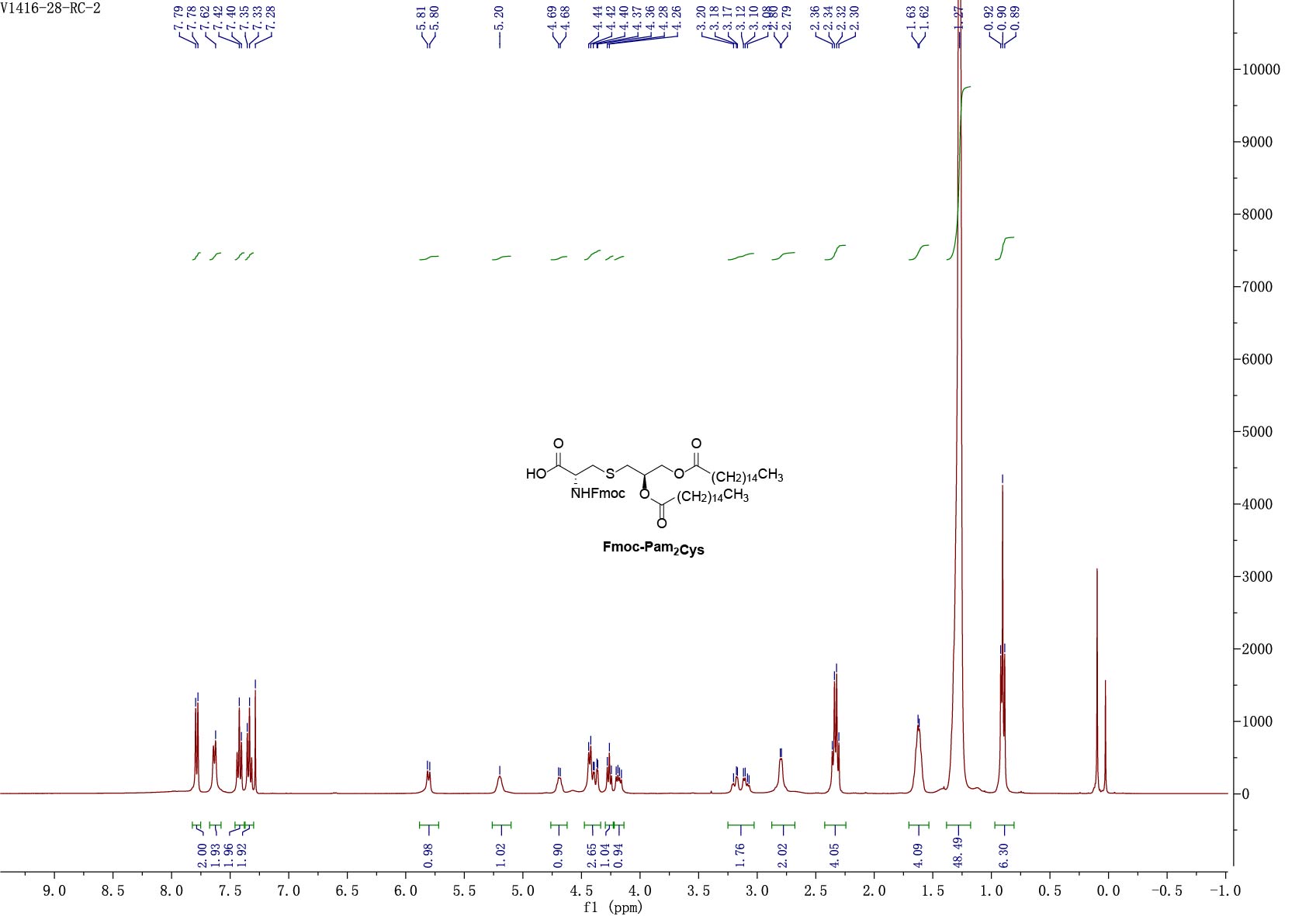
**

**Fig. S2 HPLC analysis and mass spectrometry of the novel synthetic lipopeptides**

**LP1-1: Pam2Cys-SREPKALIA**

**LP1-2: Pam2Cys-SSKGFDQPG**

**LP1-4: Pam2Cys-SADAQHFAK**

**LP1-14: Pam2Cys-SKHANNTHA**

**LP1-19: Pam2Cys-STIPSAIRH**

**LP1-20: Pam_2_Cys-SIVHEGSKY**

**LP1-23: Pam_2_Cys-SIRFVKDRT**

**LP1-24: Pam_2_Cys-SFHRAVHAY**

**LP1-30: Pam_2_Cys-SLARRNDTN**

**LP1-34: Pam_2_Cys-SHYAPLPDS**

**LP1-37: Pam_2_Cys-SRVVSKLYL**

**LP1-38: Pam_2_Cys-SLLKETAAG**

**LP1-40: Pam_2_Cys-SKVLRKHYG**

**LP2-1: Pam_2_Cys-SPALVSAER**

**LP2-2: Pam_2_Cys-SEFAHPRHG**

**LP2-3: Pam_2_Cys-SEFQATYAP**

**LP2-5: Pam_2_Cys-SHKLGLRHS**

**LP2-6: Pam_2_Cys-SAPNPSTNQ**

**LP2-7: Pam_2_Cys-SKALWANKG**

**LP2-10: Pam_2_Cys-SAVMLEQRM**

**LP2-12: Pam_2_Cys-SGGQRSPGP**

**LP2-17: Pam_2_Cys-SIKNKQSVG**

**LP2-20: Pam_2_Cys-SPNSAQFSS**

**
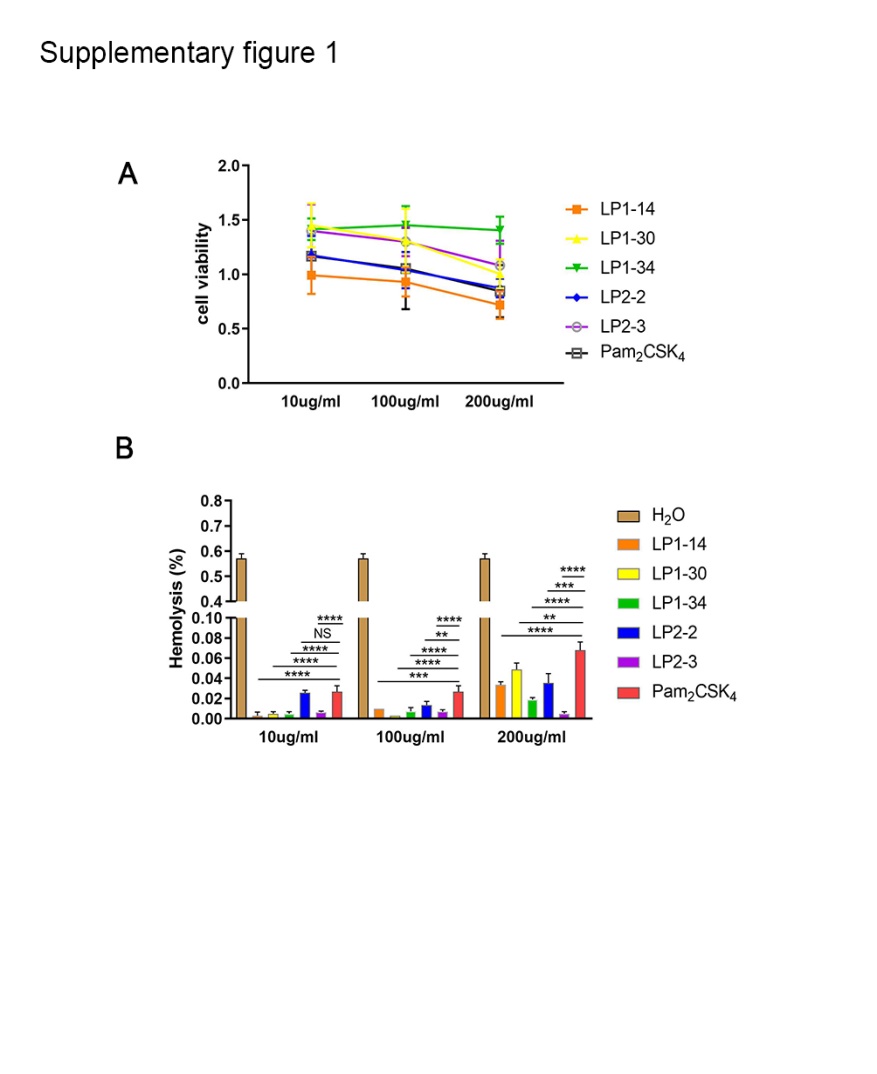
**

**Figure S3. The Safety of the lipopeptides *in vitro*.**

(A) BMDCs were treated with synthetic lipopeptides, then the cell viablility were analyzed by MTT (n=3). (B) The hemolysis was detected after red blood cells treated with synthetic lipopeptides (n=3). Data were presented as the mean ± SD. ***p*<0.01, *** *p*<0.001, **** *p*<0.0001.


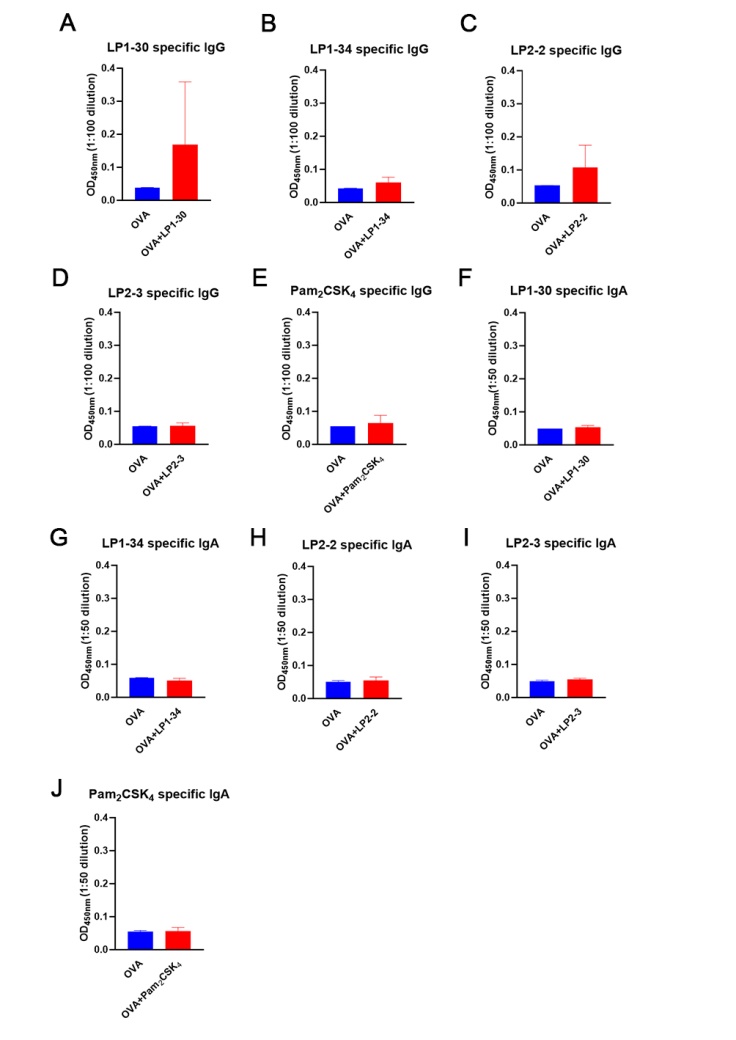


**Figure S4. Specific IgG and IgA profiles induced by each lipopeptide.**

The levels of the lipopeptide specific IgG (A-E) and IgA (F-J) in serum from mice immunized with OVA plus the novel lipopeptide were analyzed by ELISA (n=5). Data were presented as the mean ± SD.

**Table S1. The sequences of the 100 peptides generated by the Sequence Manipulation Suite**

| 1-1 | CSREPKALIA | 2-1 | CSPALVSAER |
| --- | --- | --- | --- |
| 1-2 | CSSKGFDQPG | 2-2 | CSEFAHPRHG |
| 1-3 | CSSAIASSKL | 2-3 | CSEFQATYAP |
| 1-4 | CSADAQHFAK | 2-4 | CSMADASAFA |
| 1-5 | CSVAVQGLAW | 2-5 | CSHKLGLRHS |
| 1-6 | CSPGLLSVAQ | 2-6 | CSAPNPSTNQ |
| 1-7 | CSLRERQAYG | 2-7 | CSKALWANKG |
| 1-8 | CSLWALLAQL | 2-8 | CSAVGASFLI |
| 1-9 | CSASSMYYKT | 2-9 | CSKKRMAVAA |
| 1-10 | CSGEGELALG | 2-10 | CSAVMLEQRM |
| 1-11 | CSVVGTPAAA | 2-11 | CSPVNALVFA |
| 1-12 | CSTNCVPHNE | 2-12 | CSGGQRSPGP |
| 1-13 | CSVQFERSFL | 2-13 | CSASAAKIAV |
| 1-14 | CSKHANNTHA | 2-14 | CSEDAAAEDK |
| 1-15 | CSPLGLQEAV | 2-15 | CSIQEMADNT |
| 1-16 | CSVAAAKAAE | 2-16 | CSRCILAALR |
| 1-17 | CSLPDCMGPE | 2-17 | CSIKNKQSVG |
| 1-18 | CSIDHDGPQD | 2-18 | CSLFEMIEKI |
| 1-19 | CSTIPSAIRH | 2-19 | CSIDADLALL |
| 1-20 | CSIVHEGSKY | 2-20 | CSPNSAQFSS |
| 1-21 | CSDHLNRQID | 2-21 | CSSEPAERCS |
| 1-22 | CSAGGPTAQY | 2-22 | CSKGRKGTRL |
| 1-23 | CSIRFVKDRT | 2-23 | CSIKIITSLQ |
| 1-24 | CSFHRAVHAY | 2-24 | CSLTWNASIN |
| 1-25 | CSAQAEDQNL | 2-25 | CSVAQQGLPL |
| 1-26 | CSVNHESVQL | 2-26 | CSGEEYGKVI |
| 1-27 | CSLQIPDAGR | 2-27 | CSMTALGIES |
| 1-28 | CSAMLARITD | 2-28 | CSETADLGGI |
| 1-29 | CSVTTAKNDA | 2-29 | CSLVPKVGAL |
| 1-30 | CSLARRNDTN | 2-30 | CSETILSSLA |
| 1-31 | CSICALILVM | 2-31 | CSEVLAHLYK |
| 1-32 | CSAAAGRPLA | 2-32 | CSPLLQAQDT |
| 1-33 | CSRVGDLKSI | 2-33 | CSDKDLSALY |
| 1-34 | CSHYAPLPDS | 2-34 | CSFPDIASKG |
| 1-35 | CSQGVEKTFI | 2-35 | CSAYQDISYP |
| 1-36 | CSAEIVPSVH | 2-36 | CSYYGISMRA |
| 1-37 | CSRVVSKLYL | 2-37 | CSKSSAAYLA |
| 1-38 | CSLLKETAAG | 2-38 | CSPHVTGVAR |
| 1-39 | CSIPKAGRPA | 2-39 | CSVLDLNFKL |
| 1-40 | CSKVLRKHYG | 2-40 | CSITKGLAPS |
| 1-41 | CSVLHKEESH | 2-41 | CSALALENFG |
| 1-42 | CSCTRDILIQ | 2-42 | CSPKFAEAAS |
| 1-43 | CSVFVLAKIA | 2-43 | CSVYGCAEFP |
| 1-44 | CSDAMGLIAQ | 2-44 | CSIQEKAPIG |
| 1-45 | CSAIEFTFRI | 2-45 | CSVSARTQDR |
| 1-46 | CSKHQQDGQE | 2-46 | CSGKTLAVGR |
| 1-47 | CSPRVECKVC | 2-47 | CSANPFTEMN |
| 1-48 | CSFTANFKAT | 2-48 | CSADLYPATA |
| 1-49 | CSAPEMANFM | 2-49 | CSLADCKGEL |
| 1-50 | CSWCQLAEHA | 2-50 | CSLRSLTAAA |
